# Address of Welcome

**Published:** 1899-05

**Authors:** Emory Speer

**Affiliations:** Macon, Ga.


### ADDRESS OF WELCOME.*

By Hon. EMORY SPEER,

Macon, Ga.

*Gentlemen of the Medical Association of Georgia:*

We bid you welcome to the city of our homes on the semi-centennial of your organization, not with mere ceremony, but most cordially and sincerely. While we beg you to believe that this is true, I am not a little at loss for the appropriate terms in which to express with some degree of that elaboration anticipated on such occasions, our appreciation of the honor of your coming, and our anticipations of the delight and medical erudition we will accumulate as the result of your stay. I can say nothing to further the purposes of the Association, without playing a rôle like that of Formio lecturing Hannibal on the art of war. Perhaps I should imitate the example of that most observant person, Mr. Silas Wegg, whom you will recall plumed himself on the adaptable character of his salutations. Thus, to the rector he "addressed a bow compounded of lay deference and a slight touch of shady preliminary meditation at church. To the *doctor* a confidential bow, as to a gentleman whose acquaintance with his inside he begged respectfully to acknowledge." It is, however, incumbent upon me, for our people, to make a broader though not less confidential acknowledgment. We take you, therefore, fully into our confidence when we disclose the opinion that we, here and now, welcome that profession whose conquering glories have kept even pace with the most startling and brilliant accomplishments of science, and whose votaries swiftly seize upon the latest achievements in other branches of human knowledge, and utilize them to preserve health, to lessen suffering, to prolong life, and to ameliorate the condition of mankind. I have often reflected upon the paucity, inadequacy, and, I may say, the defamatory character of a definition of the noble science of healing, given by Dr. Samuel Johnson in the nineteenth number of *The Rambler*. He had the temerity to term

* Delivered to the Georgia Medical Association in Macon, Ga., April 19th, 1899.

it "a melancholy attendance upon misery, mean submission to peevishness, and continual interruption of rest and pleasure." On the contrary, I believe it to be demonstrable that no profession has surpassed the doctor's in the enjoyment of those elevating pleasures with which a kindly Providence has blessed us, in independence and manliness of character, and in the variety of excellence with which they have, in multitudes of instances, easily outstripped the members of the other learned professions. That keen observer and critic, Dr. Samuel Parr, said: "While I allow that peculiar and important advantages arise from the appropriate studies of the three liberal professions, I must confess that in erudition, science, habits of deep and comprehensive thinking, the preeminence in some degree must be assigned to physicians." And Rousseau declared, "There is no condition which requires more study than theirs. In every country they are the most truly useful and learned of men." The truth is, that men trained in the medical profession have excelled in many departments of human endeavor. No pent-up Utica contracts their powers. Two of the most persuasive and powerful orators I ever heard were Georgia doctors, Dr. William H. Felton and Dr. H. V. M. Miller; and before this convention disperses we can all testify how well the doctors wield the "power above power of heavenly eloquence," which doth master, sway and move the eminence of men's affections.

In jurisprudence, even, they have sometimes surpassed the disciples of Blackstone who have devoted "*lucubrationes viginti annorum*" to the service of that jealous mistress. Indeed, that polished and famous commentator himself declared the medical profession to be "preeminent for general and extensive knowledge." How innumerable are the illustrations of this truth! There was Samuel Freeman Miller, for thirty years associate justice in that court for whose independence, influence and majesty, neither past nor contemporary history can afford a parallel. The first of that noble bench of jurists and statesmen accorded to the vast territory west of the Mississippi, he heard, counseled and decided with them during the most critical years of the nation's life. Of his judicial labors, more than seven hundred opinions are recorded. As a constitutional lawyer, an accurate writer has pronounced him the most eminent authority since the mighty Marshall. When he was first appointed his country was, if I may use the immortal language of Webster, "States dissevered, discordant, belligerent, a land drenched with fraternal blood"; before he died he had beheld, and largely through his own labors, the flag of reunited and peaceful America "still full high advanced, its arms and trophies streaming in their original luster, not a stripe erased or polluted, or a single star obscured." Of this great man it is well to recall that his collegiate education was had in a medical school, and that for ten years of his life, he was a country doctor in a remote county of southeastern Kentucky. Well did Chief Justice Fuller say of him: "He came with a mind trained by experience of active practice in two professions, nearly ten years in that of medicine and fifteen at the bar, a practice in either requiring for success learning, knowledge of men and things, acuteness, and, above all, the habit of decision."

But if the doctors have contributed their champions to the bar and to the bench, let it not be forgotten that the lawyers have been equally generous to the doctors. A law student under the great Story was Dr. Oliver Wendell Holmes, to become professor of anatomy and physiology in Harvard, lecturer at Dartmouth, and regular practitioner in Boston, devoted to his medical brethren, challenging with fierce defiance and exposing with keen satire what he deemed the quackery of opposing systems, and yet the gentlest of philosophers, the most buoyant of American poets, the author of the finest patriotic lyric in our language, no American writer, living or dead, exceeds him in the affections of his countrymen. We may not say how much "melancholy attendance on misery" Dr. Johnson might have read between these charming lines of this great doctor-poet:

> "Ah Clemence! When I saw thee last
> Trip down the Rue de Seine,
> And turning when thy form had passed,
> I said, 'We meet again'"; nor what "continual interruption of rest and pleasure" in his rollicking stanzas on a punch bowl:

"'I tell you, there was generous warmth in good old English cheer;
I tell you, 'twas a pleasant thought to bring its symbol here;
'Tis but the fool that loves excess; hast thou a drunken soul?
The bane is in thy shallow skull, not in my silver bowl!"

What "mean submission" is there in that inspiring strain, "Old Ironsides," whose martial fire will inflame as long as succeeding generations shall behold that "banner in the sky" and recall how "beneath it rung the battle's shout and burst the cannon's roar?" And, moreover, the doctors have not been content to inspire others to deeds of heroic renown. They have themselves fought the enemies of their country with all the valor of the Crusaders of old who swarmed up the breach of Ascalon. Such a one was Dr. Levasseur of the French Revolution. Carlisle tells us he was of small stature, as hard as flint which also carries fire in it, by trade a mere pacific surgeon-accoucher, he has mutinies to quell, mad hosts bellowing far and wide. Far in the afternoon he declares that the battle is not lost, that it must be gained; and fights, himself, with his own obstetric hand; horse shot under him, on foot, "up to the haunches in tide water" cutting stoccado and passado there in defiance of water, earth, air, fire. There is "mean submission" for you!

Nor need we cull our illustrations from foreigners alone, or from the distant past. What pathetic form is that bending over the wounded marine on the missile-swept hill of Guantanamo presently shot through the brain to sink in death on the prostrate form of his comrade? Dr. John Blair Gibbs, surgeon on duty. What young and manly figure do we behold in the stifling and bloody jungle at Guasimas, under the sheeted volleys of the Spaniards, unmindful of self, now ministering to the dying as the life-blood ebbs away, now bearing the wounded on his broad shoulders to shelter? Dr. Robb Church, surgeon of the Rough Riders, and grandson, we think, of Alonzo Church, long president of our own University, close kinsman of that other Church whose stainless sword flashed on many a stricken field, as he followed the guidons Stuart and Hampton. And what heroic officer is this, who, pacing the fiery crest of battle, holds his men to their deadly work, sweeps with them through the barbed entanglements, the leaden hail from machine-guns and Mausers, and plants the "flag of the free man's home and hope" on the gory ridge of San Juan? Dr. Leonard Wood.

It is, however, in his own daily professional life that the physician is most captivating to the sensibilities and appreciation of mankind. Even the cynical Voltaire exclaimed, "The man who is occupied in restoring health to his fellows is far above all the grandees of earth. He belongs to the divinity." It would be unprofitable, did time permit, for me to enlarge upon a truth to which all sentient minds render ready and grateful accord. Nor may I trust myself to speak, and mine is but the common experience, of the succor and support and blessings bestowed by knightly brothers, living and dead, of this more than golden company, in those hours of affliction which are common to man, for the sources of grateful memory lie hard by the fountain of tears. It is the brotherhood of which I speak. Noblemen, heroes, benefactors all. They pale not before Pestilence's horrid form.

"The bloodless stabber calls by night,
Each answers, 'Here am I.'"

It is indeed a heroic army.

"Along its front no sabres shine,
No blood-red pennons wave,
Its banner bears the single line,
'Our duty is to save.'"

Here in this typical city of Georgia, where, fifty years ago, venerable and venerated men now present witnessed the auspicious birth of this your beneficent organization, gentlemen of the Georgia Medical Association, you are welcome in our homes and our hearts, and long may you survive to enjoy the love and gratitude you have so richly won.

